# Editorial: mRNA Translational Control as a Mechanism of Post-transcriptional Gene Regulation

**DOI:** 10.3389/fmolb.2022.947516

**Published:** 2022-06-23

**Authors:** Daniel L. Kiss, Deepika Vasudevan, C. Kiong Ho, Neva Caliskan

**Affiliations:** ^1^ Center for RNA Therapeutics, Weill Cornell Medical College, Houston Methodist Research Institute, Houston, TX, United States; ^2^ Department of Cell Biology BST, University of Pittsburgh, Pittsburgh, PA, United States; ^3^ Faculty of Medicine, University of Tsukuba, Tsukuba, Japan; ^4^ Medical Faculty, Helmholtz Institute for RNA-based Infection Research (HIRI-HZI), University of Würzburg, Würzburg, Germany

**Keywords:** mRNA translational control, RNA binding proteins, noncanonical translation factors, ribosome frameshifting, translation initiation factors, ribosome profiling, bacterial translation, untranslated regions (UTRs)

## Introduction

Precise control of gene expression is central for every organism. Much research has been done to uncover the network of regulatory pathways controlling the RNA life cycle and how RNA levels change rapidly to modulate the amount of proteins made by the cells. In principle, every point in the life cycle of an mRNA from its transcription, modification, splicing, processing, trafficking, translation, and decay is monitored by one or more RNA surveillance mechanisms. Together, these regulatory mechanisms maintain normal cellular activity and provide timely responses to adapt to changing environments such as during starvation, differentiation, or infections. This special section presents a collection of recent work in prokaryotic and eukaryotic translational control and the role of RNA elements and modifications in regulation of translation.

The contributions to this special section fell into three distinct groups. The first of these groups include three reviews examining the contributions of translational control to stem cells, a second review that helps us better understand the translational impact of RNA binding proteins (RBPs) and microRNAs, and a third review examining ribosome frameshifting. In the first paper, Wang and Amoyel describe recent advances that demonstrate the outsized role of mRNA translational regulation in stem cell proliferation and differentiation. They survey how bulk and mRNA selective translation events dictate stem cell fate and describe translation regulatory mechanisms that affect stem cell specification across many types of stem cells. This is critical as most multicellular life originates from a single totipotent cell that divides and differentiates, giving rise to an entire organism. Next, Jungers and Djuranovic review the literature describing how RBPs and miRNAs act synergistically or antagonistically to regulate mRNAs via 3′UTR sequences. We’ve known that interactions between trans-acting regulatory factors on individual mRNAs create an elaborate landscape of effects that regulate genes post-transcriptionally. They close by highlighting the implications of these regulatory mechanisms on human disease, particularly cancers. Lastly, many viral, bacterial, and some eukaryotic RNAs contain sequence elements that shift ribosomes to alternative reading frames during translation. Riegger and Caliskan discuss the current knowledge regarding programmed ribosomal frameshifting. They summarize the role(s) of RNA secondary structures and highlight how *trans*-acting RNA modulators can dynamically adjust the timing and efficiency of frameshifting events. Ribosome frameshifting provides new targets for disrupting viral translation strategies and presents opportunities to impact infections and immune responses.

In the second group of papers, three groups use prokaryotic systems to ask fundamental questions about translational control. High-resolution structures of active ribosomes are critical for understanding translational regulation. Belinite et al. successfully purified stable 30S small ribosome subunits from *S. aureus* and solved their structures complexed with RNA by cryo-electron microscopy in the presence and absence of spermidine. Remarkably, spermidine stabilizes helix 44 to form the active conformation. This study provides important insights into how ribosome structure could be regulated during translation. Next, we’ve known that certain archaeal transcripts, including tRNA, rRNA, and sRNA, undergo post-transcriptional maturation, often involving a circular RNA intermediate. However, the mechanism underpinning selective circularization occurs was unknown. Liu et al. demonstrated that ATP-dependent RNA ligase (Rnl) generates circular C/D Box sRNAs and validated the structural features required for circularization. Interestingly, their work suggests that, either directly or indirectly via C/D Box sRNA circularization, Rnl also contributes to rRNA processing near the helix 98 region of 23S rRNA. Finally, the anti-Shine-Dalgarno (anti-SD) sequence is a highly conserved rRNA sequence that hybridizes with the Shine-Dalgarno (SD) sequence upstream of the start codon to promote translation initiation. However, species in the phylum Bacteroidota have very few mRNAs with consensus SD sequences. McNutt et al. used comprehensive mutation analysis of anti-SD sequence in *E.coli* and *F. johnsoniae* to gain new insights into the mechanism of SD-independent translation. Together, these three works leverage studies investigating ribosome structure, small RNA processing, and the impact interactions of ribosomal and mRNA sequences to expand our knowledge of prokaryotic translational regulation.

The final group of research articles detail new findings that describe the contributions of RNA modifications and sequences to translational control. Douka et al. describe Ribo-seq approaches tailored for human and Drosophila melanogaster cell lines. They illustrate that even subtle changes in experimental conditions can alter overall quality of ribosome footprinting. Furthermore, the authors show the varying impact of antibiotic pretreatment on different cell types. Overall, this study highlights key attributes that require careful optimization in each new system to obtain high-quality ribosome profiling data. Second, using budding yeast mutants and ribosome profiling Stanciu et al. show that eIF3’s (eukaryotic initiation factor 3) contributions were often mediated by sequences in 5′ untranslated regions. eIF3 is a key initiation factor that functions throughout translation initiation including during mRNA recruitment and has been linked to the selective translation of specific classes of mRNAs. Third, IF4B binds and recruits mRNA to preinitiation complexes. Liu et al. disrupted yeast eukaryotic initiation factor 4B (eIF4B) and 40S binding motifs to evaluate how these factors impact adaptation and survival in diverse cellular environments. The authors show that N-terminal domain deletions of eIF4B leads to decreased translation rates, especially on mRNAs with long and structured 5′ UTRs. This work suggests that the NTD of eIF4B affects the translation of a subset of mRNAs, thereby impacting cellular stress responses. Finally, RNA modifications such as N6 methyl-adenosine (m6A) influence post-transcriptional gene regulation. IGF2BP2 (insulin-like growth factor 2 mRNA-binding protein 2) is an RNA binding protein and m6A reader known to regulate the localization, translation, and stability of mRNAs. In their work, Han et al. demonstrate that IGF2BP2 regulates the translation of ATG12 (autophagy-related 12) mRNA indirectly through the MALAT1 lncRNA.

## Summary

Overall, these articles highlight the impact of RNA-based regulatory events during translation. These articles also shed light on the interplay of protein factors and mRNA elements that fine tune the dynamics of translation. These aspects will be relevant to understanding the complexity of the gene expression networks and their impact on cellular response in cell proliferation, cell fate and immune regulation.

